# Infective Endocarditis Caused by Corynebacterium striatum: Navigating Challenges and Treatment Strategies in an Emerging Threat

**DOI:** 10.7759/cureus.49526

**Published:** 2023-11-27

**Authors:** Zied Gaifer, Basim S Samman, Nouf A Albluwi

**Affiliations:** 1 Internal Medicine, Ministry of National Guard - Health Affairs, Prince Mohammed Bin Abdulaziz Hospital, Medina, SAU; 2 Internal Medicine, Sulaiman Al-Rajhi University, Medina , SAU

**Keywords:** linezolid, transesophageal echocardiography, vancomycin, infective endocarditis, corynebacterium striatum

## Abstract

*Corynebacterium striatum *is a type of bacteria normally found in the environment and is considered a benign microbe on the human body surface. However, it can induce severe medical conditions, including bacteremia, infective endocarditis, osteomyelitis, and infections, in other organs. This case study focuses on a 56-year-old male patient with multiple comorbidities who presented with an ischemic stroke. Several days after the insertion of a right internal jugular line, the patient developed fever and tachycardia. Blood cultures revealed the presence of *Corynebacterium striatum*, a Gram-positive bacilli. Transesophageal echocardiography confirmed the diagnosis of complicated infective endocarditis (IE) with mitral valve vegetation and moderate mitral regurgitation. Prompt treatment with appropriate antibiotics, including linezolid and later vancomycin, led to the patient's improvement and eventual discharge in good condition. This case highlights the importance of early recognition, aggressive management, and accurate diagnosis in cases of IE caused by *Corynebacterium striatum*. Proper antibiotic selection is crucial, considering the emerging antibiotic resistance patterns associated with this pathogen. By addressing these aspects, patient outcomes can be improved, and potential complications such as IE can be prevented.

## Introduction

Corynebacterium, a group of Gram-positive rods consisting of over 100 species, has been associated with medical relevance [1]. While non-diphtheria Corynebacterium species were traditionally considered part of the human normal flora in the skin and mucous membranes, recent reports have identified them as opportunistic pathogens [1].

Usually, the presence of Corynebacterium in blood cultures is considered as contamination. However, it has recently been found to cause infections in both immunocompromised and immunocompetent individuals [2]. Studies investigating different patient cohorts have indicated that a significant percentage, ranging from 44% to 71%, of those with positive cultures of Corynebacterium have true infections [3].

## Case presentation

A 56-year-old male patient was transferred to our hospital after spending five weeks at a previous medical facility, where he was initially admitted for an ischemic stroke. The patient's medical history included type 2 diabetes mellitus, hypertension, chronic kidney disease, and a stroke.

Upon admission, the patient was alert with a Glasgow Coma Scale score of 14/15. He demonstrated motor deficits, with his left upper and lower limbs having 1/5 strength and his right upper and lower limbs having 3/5 power.

A right internal jugular line was inserted to initiate hemodialysis due to worsening kidney function, indicated by increasing creatinine and BUN levels. Additionally, the patient became anuric and did not improve with conservative medical management.

After five days of having the right internal jugular line in place and two days into his general ward stay, the patient experienced tachycardia (120 beats per minute) and a fever (38.1°C). The patient's blood pressure was 131/87 mmHg, and he remained dependent on 2 L of supplemental oxygen administered through a nasal cannula.

The patient denied experiencing any symptoms such as neck pain, headache, photophobia, phonophobia, cough, palpitations, chest pain, diarrhea, vomiting, abdominal pain, dysuria, skin rash, or joint pain. The physical examination revealed no notable findings apart from his feverish condition.

A comprehensive diagnostic investigation was initiated, including the collection and analysis of blood, urine, and respiratory samples for the presence of infectious agents. He was initially prescribed piperacillin/tazobactam based on empirical evidence. Routine laboratory tests and tests for inflammation indicated elevated levels of white blood cells (12.9×10^9^/L), neutrophils (10.40 ×10^9^/L), neutrophil percentage (80.70%), and C-reactive protein (128.4 mg/L) (Table 1). The blood culture results subsequently indicated the presence of *Corynebacterium striatum*, isolated from both aerobic and anaerobic vials. The urine culture showed identical *Candida albicans* results as previously observed. The respiratory culture results were normal, and a follow-up chest X-ray showed a reduction in the area of collapsed lung tissue in the lower left region, with no new signs of infection.

**Table 1:** Inflammatory markers and basic laboratory results during hospital stay. CRP: C-reactive protein, Hgb: hemoglobin, WBC: white blood cell, BUN: blood urea nitrogen.

Lab parameters (reference range)	Time of admission	Time of blood culture	Day culture came negative	Day of discharge
CRP (0.1–4.9 mg/L)	59.3	128.4	45.1	63.6
Hgb (135–180 g/L)	89	65	60	95
WBC (4–11×10^9^/L)	10.6	12.9	8.4	10.0
Basophil# (0–0.1×10^9^/L)	0.06	0.07	0.10	0.12
Basophil% (0–3 %)	0.59	1.06	1.12	1.24
Monocyte# (0.1–1.1×10^9^/L)	1.23	0.92	0.96	0.81
Monocyte% (3–9 %)	11.6	13.60	11.4	8.13
Eosinophil# (0.1–0.7×10^9^/L)	0.22	0.07	0.21	0.22
Eosinophil% (1–6%)	2.02	1.08	2.47	2.23
Neutrophil# (2–7.5×10^9^/L)	7.33	10.40	5.47	7.08
Neutrophil% (36–75%)	68.90	80.70	64.90	70.90
Lymphocyte% (20–45%)	16.90	15.40	19.6	17.50
Lymphocyte# (1–4.4×10^9^/L)	1.80	1.03	1.65	1.75
Platelet (150–450×10^9^/L)	200	136	287	212
Creatinine (64–110 µmol/L)	670	387	431	175
BUN (3–9.2 mmol/L)	42.9	17.4	15.1	9.0

After stopping the initial antibiotics and removing the central line, subsequent blood cultures from both the peripheral and central lines consistently detected *C. striatum*. After consulting with the infectious disease team, the patient was prescribed linezolid at 600 mg orally, twice daily.

Transesophageal echocardiography was advised to exclude the possibility of infective endocarditis, and it detected a small growth on the atrial side of the mitral valve leaflet, most likely vegetation, accompanied by moderate mitral regurgitation.

After five days of line removal and linezolid treatment, peripheral blood cultures returned negative results, and inflammatory markers started to decline. The patient maintained a course of linezolid for 14 days, after which they transitioned to intravenous vancomycin due to improved renal function. After undergoing a four-week treatment with vancomycin, he was released in a satisfactory overall state, with instructions to continue taking linezolid orally for an additional 21 days.

Five months after being discharged from the hospital, the patient returned to the outpatient clinic for a check-up and reported no symptoms. Prescriptions were given to refill medications for his ongoing medical conditions, which included diabetes mellitus, hypertension, and chronic kidney disease. Three months later, the patient showed up for a clinic visit, confirming his stability.

## Discussion

*C. striatum*, a gram-positive, rod-shaped bacterium, is found in the environment and is a component of the normal microbial population on the surface of the human body [1].

Clinically, it is commonly considered nonpathogenic and a specimen contaminant. However, there have been rare instances of it being associated with opportunistic infections, especially in hospital settings [4]. It has the potential to induce severe medical conditions, including bacteremia, infective endocarditis, osteomyelitis, and infections in other organs [5].

In specific patient populations, *C. striatum* has become an infectious agent. These populations include immunosuppressed patients and those with a history of invasive procedures, prosthetic medical devices, and intravascular access [6]. The recent increase in *C. striatum* infections can be ascribed to various factors, such as the burgeoning population of immunocompromised individuals, the broader utilization of invasive medical devices, longer hospital stays, and extended antibiotic exposure [2].

It can be challenging to distinguish between an actual infection and colonization from patient specimens. To differentiate, it is crucial to repeatedly identify the organism, isolate it from a sterile site, and maintain a high level of clinical suspicion in the appropriate host, especially in cases involving immunocompromised individuals or medical devices [7].

When considering other bacterial pathogens linked to endocarditis, Corynebacterium is comparatively rare [8]. Endocarditis caused by *C. striatum* commonly arises in individuals with predisposing factors, including prosthetic heart valves, a history of invasive medical procedures, or intravascular devices. However, it can also manifest in cases involving native valves [9]. Like other bacteria, the mortality rate linked to corynebacterial endocarditis ranges from 31% to 43% [9,10]. It is worth mentioning that endocarditis caused by *C. striatum* has the highest probability of survival compared to other types of Corynebacterium endocarditis [11].

*C. striatum* possesses unique characteristics that differentiate it from other members of the Corynebacterium genus, making it capable of causing endocarditis. *C. striatum* can create biofilms on medical devices, aiding its survival and resistance to removal. This is particularly true for prosthetic heart valves or intravascular implants [12]. In addition, *C. striatum* frequently exhibits resistance to a broad range of beta-lactam antibiotics, rendering conventional treatment options less effective [13]. This heightened resistance can pose challenges in treating infections caused by this organism, such as endocarditis [4].

*C. striatum* is well known for its resistance to antibiotics, especially the penicillin class [13]. IV vancomycin has been suggested as a treatment choice. However, there have been reports of vancomycin resistance, possibly due to extended bacterial exposure to antibiotics in hospital settings [14].

Effective management of *C. striatum* infections is complicated by the absence of universally recognized criteria for selecting antibiotics. Recent research points to an alarming trend toward increased multidrug resistance, although earlier studies suggested susceptibility to a wide range of antibiotics. Nevertheless, vancomycin has proven effective against *C. striatum* infections, as evidenced by its low minimum inhibitory concentration. Our case demonstrates that the strain was resistant to multiple antibiotics but still susceptible to linezolid and vancomycin, which guided our treatment strategy.

## Conclusions

To summarize, *C. striatum* is a newly recognized pathogen that primarily impacts individuals who are susceptible due to the presence of medical devices or a history of invasive procedures. Once considered harmless, it now presents a danger by causing severe conditions such as infective endocarditis. Challenges include distinguishing between infection and colonization, combating significant drug resistance, and addressing the formation of biofilms on medical devices.

The complexity of managing *C. striatum* infections is highlighted in our case, as the increased multidrug resistance further complicates antibiotic selection. A favorable outcome was achieved through prompt and multidisciplinary intervention, involving the removal of the central line and the administration of tailored antibiotic therapy. The mortality risk, while significant, underscores the necessity of timely diagnosis and proper treatment. Given the increasing clinical importance of *C. striatum*, it is imperative to conduct additional research to develop practical treatment approaches, gain insight into the factors contributing to its virulence, and implement preventive measures to address the growing number of infections associated with this organism.
